# Endoscopic retrograde cholangiopancreatography in the elderly: results of a retrospective study and a geriatricians’ point of view

**DOI:** 10.1186/s12876-018-0764-4

**Published:** 2018-03-14

**Authors:** Marianna Galeazzi, Paolo Mazzola, Breanna Valcarcel, Giuseppe Bellelli, Marco Dinelli, Giulio Maria Pasinetti, Giorgio Annoni

**Affiliations:** 1 University of Milano-Bicocca, School of Medicine and Surgery, U8 Building, Floor 4, Lab 4045, Via Cadore, 48 - 20900, Monza, MB Italy; 2 NeuroMI – Milan Center for Neuroscience, Clinical Neurosciences research area, Milan, MI Italy; 3 San Gerardo Hospital ASST Monza, Acute Geriatrics Unit, Monza, MB Italy; 4 San Gerardo Hospital ASST Monza, Endoscopy Unit, Monza, MB Italy; 5 Icahn School of Medicine at Mount Sinai, New York, NY USA; 6 James J Peters Veterans Affairs Medical Center, Bronx, NY USA

**Keywords:** Endoscopic retrograde cholangiopancreatography, ERCP, Endoscopy, Elderly, Procedural complications

## Abstract

**Background:**

The incidence of biliary tract pathology is growing with an age-related trend, and progresses as the population ages. Endoscopic Retrograde Cholangiopancreatography (ERCP) represents the gold standard for treatment in these cases, but evidence about its safety in the elderly is still debated.

**Methods:**

We retrospectively analyzed the clinical records of all patients aged ≥65 undergoing ERCP between July 2013 and July 2015. Of 387 ERCP cases, 363 (~ 94%) were completed entirely. The mean age of the study population (*n* = 363) was 79.9 years old (range 70–95), with 190 subjects aged 70–79 and 173 older than 80. We recorded demographics, Charlson Comorbidity index (CCI), American Society of Anesthesiologists (ASA) physical status classification score, indication for the use of the ERCP procedure, and clinical outcomes. Then, we tested all variables to identify the potential risk factors for complications associated with the procedure.

**Results:**

The older group (those ≥80 years old) showed significantly more patients with ASA Classes III-IV than the younger one (those ≤79 years old). Interestingly, the CCI was higher in the younger group (*p* = 0.009). The overall complication rate was 17.3% without inter-group differences. Older age, sex, CCI and intra-ERCP procedures were not related to a higher risk of complications, and the multivariate regression did not identify any of the considered variables to be an independent risk factor for complications.

**Conclusion:**

ERCP appears as safe in the patients aged 80 years and older, as it is in those aged 70–79 years old in our study, however, a selection bias may affect these findings. A study including a comprehensive geriatric assessment will contribute to shedding light on this issue.

## Background

The incidence of biliary tract pathology is growing due to the overall aging of the population worldwide, and this is particularly evident among the number of those over the age of 80^1^. Endoscopic Retrograde Cholangiopancreatography (ERCP) represents the gold standard exploratory technique for the treatment of biliary or pancreatic tract pathology, and it could be often performed with therapeutic intent by realizing procedures such as insertion of bile duct stents and/or endoscopic sphincterotomy. However, evidence about its safety in the elderly is still controversial.[1–5] A recent systematic review by Day et al.[4] explored the issue of effectiveness and safety of ERCP in octogenarians (i.e. ≥80 years old) versus younger seniors and late adults, showing that perforation, hemorrhage, biliary infection, and other main complications of the procedure were not statistically different among the three groups. However, despite the sample size of the meta-analysis, patients did not undergo a comprehensive approach to be characterized. Instead of more inclusive tools, the frequency of symptoms, the proportion of some comorbid conditions (namely hypertension, diabetes, coronary heart and cerebrovascular diseases, and “others”), and the American Society of Anesthesiologists (ASA) physical status classification score were the parameters collected.[6] From a geriatric perspective, the assessment of older patients is an interdisciplinary process that should include multiple domains, such as functional and nutritional status, cognition, accurate evaluation of comorbidity, and many others[7].

In order to add knowledge to this scenario and to understand if this gap in patient’s assessment applies to our population, we retrospectively analyzed the clinical records of older patients undergoing ERCP in the endoscopy unit of an Italian university hospital. Our aim was to assess how the specialists in endoscopy evaluate patients undergoing ERCP, the success rate of this procedure, and the incidence of complications in octogenarians compared to that of younger seniors (aged < 80 years old).

## Methods

### Study design and subjects

This retrospective observational single-center study was carried out at San Gerardo Hospital ASST Monza, a large Italian university hospital. We retrieved data from the Endoscopy Unit electronic registry, collecting all the ERCP procedures that took place from July 1st, 2013 to July 31st, 2015 in an electronic spreadsheet. We considered only ERCPs performed in patients ages 65 and older, and divided the study population into two groups according to the age criterion (65–79 vs. ≥80).

All data were de-identified according to the Italian legislation “Data Protection Code – Legislative Decree no. 196/2003” (available at http://www.garanteprivacy.it/home_en/italian-legislation) for the protection of personal information. Permission for the use of this information was granted by a specific consent form signed by each patient or his/her legal representative for undergoing the endoscopy procedure. The informed consent forms are stored in the archive of San Gerardo Hospital ASST Monza (Monza, Italy).

For every ERCP procedure performed, we collected the following information: patient’s age and gender, comorbid conditions, date, indication and duration of the procedure, ASA physical status classification score, [6] duration of sedation/anesthesia, additional procedures, complications, success in addressing the indications, diagnosis, and whether the ERCP was completed or not. Multiple procedures performed in the same patient were also taken into account.

With more detail, the ASA score is a qualitative system originally developed to describe the pre-operative fitness of patients scheduled for surgical procedures. It currently consists of 6 classes, from ASA 1 (normal healthy subject) to ASA 6 (a declared brain-dead patient). The higher the ASA class, the poorer the patient’s health status is considered to be. Comorbid conditions were assessed with the Charlson Comorbidity Index (CCI), [8] a weighted index that assigns a different risk score (from 1 to 6) to 22 conditions and their related relative risk of 1-year mortality. The higher the CCI score, the higher the risk of 1-year mortality.

### Procedures

All the ERCP procedures were performed by four experienced gastroenterologists with 10 or more years of expertise in endoscopy and in this procedure specifically.

Patients underwent sedation as indicated in our Endoscopy Unit sedation protocol, which agrees with national guidelines, available at: http://www.sied.it/files/12ConigliaroFantiGIED3_14.pdf; http://www.sied.it/files/Lineeguidaperlasedazioneinendoscopiadigestiva.pdf .

It should be considered that regulations limit the drugs that non-anesthetists could administer to meperidine or fentanest and midazolam. In ASA I and II patients who do not require a urgent procedure, the endoscopic team performed moderate sedation with opioids and midazolam. Sedation is constantly monitored as it is used for other endoscopic procedures.

ASA III/IV patients and those who need to undergo urgent ERCP were sedated and monitored by an anesthetist who decided to perform deep sedation or general anaesthesia according to its judgement and the subject’s clinical conditions at the moment of undergoing the procedure. All urgent ERCPs were performed in the operating room irrespectively of the type of ERCP and sedation chosen.

### Definition of complications

The adverse events we recorded during and/or in response to ERCP procedures were defined as follows:

- Pancreatitis: the onset of new abdominal pain with at least a three-fold elevation of serum amylase or lipase levels, at least 24 h after the procedure;

- Perforation: retroperitoneal or bowel-wall perforation, as evidenced by imaging techniques;

- Bleeding: clinical evidence of hemorrhage, with a decrease in hemoglobin > 2 g/dL or the need for endoscopic or transfusion treatment;

- Cholangitis: temperature of more than 38 °C for 24–48 h occurring after the procedure and thought to have a biliary cause, without evidence of concomitant infection;

- Cardiopulmonary adverse events: myocardial infarction, cerebrovascular accident, congestive heart failure, cardiac/respiratory arrest, arrhythmia, hypoxemia (oxygen saturation < 90%), hypotension (systolic blood pressure < 90 mmHg), bradycardia (heart rate < 60 beats/min), tachycardia (heart rate > 110 beats/min), or vasovagal response, all of which were attributed to the performance of an ERCP procedure;

- Mortality related/not related to the performance of the ERCP: death within 48 h after a procedure with complications or incomplete endoscopic treatment (for example, relief of bile duct obstruction was not achieved by the intervention) was regarded as a procedure-related death.

These complications were defined by Cotton’s criteria [9].

### Outcomes

After describing the parameters and information we were able to collect from the hospital registry, the primary outcome was to assess the incidence of complications or deaths associated with ERCPs.

The secondary outcome was the identification of the baseline characteristics and ERCP-related variables able to predict the onset of complications.

### Statistical analysis

All analyses were performed with the software SPSS Version 23.0 (IBM Corp., Armonk, NY, USA). Continuous variables were expressed as mean ± standard deviation (SD). Categorical variables were expressed as frequencies and percentages. Comparisons between the study groups were performed with the Student’s t-test for continuous variables and the Chi-square test for categorical variables. All the comparisons were two-tailed.

The analyses of the risk factors for ERCP-related complications were performed through univariate and multivariate logistic regression models. Significance was obtained for values of *p* < 0.05.

## Results

### Patients’ characteristics

Seven hundred and thirty-two ERCPs were performed at San Gerardo Hospital from July 1st 2013 to July 31st 2015. Of them, 485 (66.3%) were performed in patients ages 65–95. To date, 9% (33 subjects) of the population was nonagenarian (i.e. ≥90 years old). Four hundred and sixty-five patients were hospitalized at San Gerardo Hospital, while 20 subjects were admitted into other nearby hospitals but underwent ERCP in our Endoscopy unit; 53 ERCPs were considered as urgent procedures.

Ninety-seven patients underwent more than one procedure for technical reasons (e.g.: stent placement and removal) or for treatment of a second recurrence of a common bile duct (CBD) stone. Specifically, one patient underwent seven ERCPs, three patients underwent five ERPCs, eight patients underwent four ERCPs, 34 underwent three procedures, and 51 underwent ERCP twice.

Interestingly, the CCI was significantly higher in the younger group compared to that of the older group (Table 1). However, considering the age-adjusted CCI, we observed the opposite situation with the CCI being higher in the older group than that of the younger one, but this difference did not reach statistical significance.

**Table 1:** Baseline characteristics of the enrolled patients

Groups	Age 65–79 *N* = 280	Age ≥ 80 *N* = 185	All *n* = 465	*p*-value
Mean age, years	72.78 ± 4.4	85.29 ± 4.18	77.6 ± 7.64	< 0.001
Gender, n (%)				< 0.001
Male	146 (52.1)	66 (35.7)	212	
Female	134 (47.9)	119 (64.3)	253	
Charlson Comorbidity Index (CCI), mean	1.6 ± 2.0	1.2 ± 1.4	1.2 ± 1.8	0.008
Age-adjusted CCI, mean	4.4 ± 2.0	5.3 ± 7.0	4.8 ± 1.8	0.059
ASA score, n				0.048
I	2 (0.7)	–	2	
II	210 (75.0)	113 (61.1)	323	
III	63 (22.5)	69 (37.3)	132	
IV	5 (1.8)	3 (1.6)	8	
V	–	–	–	
Urgent ERCP, n (%)	27 (9.6)	26 (14.1)	53 (11.6)	0.179

Data are presented as mean ± SD or as a number and percentage. *ASA* American Society of Anesthesiologists

The ASA scores, considering each separate class, presented significant differences between the groups (*p* = 0.048). We also grouped the ASA scores between classes I-II (low intra-operative risk) and classes III-IV (moderate-to-high intra-operative risk), rather than all four classes individually, and we observed that the older group had a significantly greater proportion of class III-IV ASA scores (38.9% vs. 26.7% of the younger group, *p* = 0.011).

Table 2 shows that the main indication for ERCP intervention was CBD stones, followed by stent placement/removal. No statistical difference between the groups was found.

**Table 2:** Indications for ERCP procedures in the study groups and in the population as a whole

Indication	Age 65–79 N = 280	Age ≥ 80 N = 185	All *N* = 465
CBD stones/ biliary colic	92 (32.9)	74 (40.0)	166 (35.7)
Stenosis	51 (18.2)	28 (15.1)	79 (17.0)
Acute Pancreatitis	16 (5.7)	12 (6.5)	28 (6.0)
Stent placement or removal	66 (23.5)	40 (21.6)	106 (22.8)
Cholangitis	40 (14.3)	29 (15.7)	69 (14.8)
Other	15 (5.4)	2 (1.1)	17 (3.7)

All indications are reported as a number and percentage (%)

#### ERCP: Procedures, success rate and complications

Among a total of 465 procedures, 438 were completed: 265 ERCPs belonged to the younger group, 173 to the older one. The rates of success of ERCP in the younger and older group were 94.6% and 93.7%, respectively.

The 27 failures were due to complications (3/27: 2 bradycardia and 1 perforation), single diverticulum associated ampulla (17/27), altered surgical anatomy (3/27), and multiple large diverticula with no identification of the Vater ampulla (4/27).

All procedures performed during the ERCP and the diagnoses are presented in Table 3 and Table 4, respectively. No statistical differences between the groups were found. CBD stones were the most frequent diagnosis.

**Table 3:** Intra-ERCP procedures in the study groups and in the population as a whole. All procedures are reported as a number and percentage (%)

Procedures	Age 65–79 *N* = 280	Age ≥ 80 *N* = 185	All *N* = 465
Sphincterotomy, n %	74 (28)	47 (27)	121 (27.6)
Stent placement or removal, n %	101 (38)	65 (37.5)	166 (37.9)
Sphincterotomy and stent placement, n %	85 (32)	59 (34)	144 (32.8)
Other, n %	5 (2)	2 (1.5)	7 (1.7)
Incompleted procedure	15 (5.3)	12 (6.4)	27 (5.8)

**Table 4:** ERCP diagnoses in the study groups and in the whole population. All diagnoses are. reported as number of patients and percentage (%)

Diagnosis	Age 65–79 *N* = 265	Age ≥ 80 *N* = 173	All *N* = 438
CBD stones	119 (44.9)	87 (50.3)	206 (47.0)
Stenosis	50 (18.9)	31 (17.9)	81 (18.5)
Malignant stenosis	40 (15.1%)	31 (17.9%)	71(16.2%)
Inflammatory stenosis	10 (3.8%)	0	
Stent placement or removal	73 (27.6)	49 (28.3)	122 (27.9)
Other	22 (8.3)	6 (3.5)	28 (6.4)

*CBD* common bile duct

Fifty-three complications were related to the procedure (Table 5). In the younger group, the complication rate was 13.2% vs. 10.0% in the older one.

**Table 5:** ERCP-related complications

Complications	Age 65–79	Age ≥ 80	All
Cholangitis-Septic cholangitis	17	6	23
Cardiopulmonary	6	3	9
Bleeding	4	0	4
Acute Pancreatitis	5	4	9
Perforation	3	2	5
Death ERCP related	2	1	3
Total ERCP-Related complications	**37**	**16**	**53**

All complications were higher in the younger group. Two out of 4 patients who had bleeding (younger group) used to take aspirin. To date, no one used oral anticoagulants.

Seven complications (13%) happened during urgent ERCPs, six in the younger group (four sepsis, two perforations), and one in the older group (sepsis). No statistical difference between these groups emerged.

Ten episodes were considered as not related to ERCP: fever without sepsis (eight patients), ischemic stroke (n = 1), and diarrhea (n = 1).

Nine patients died after the ERCP procedure, three of whom due to ERCP-related shock. Those cases underwent ERCP for CBD stones and for stent removal, and presented only cardiovascular comorbidities.

Six patients, of the nine who died, passed because of shock from septic cholangitis, despite successful completion of the ERCP. Their ages were 68, 69, 79, 89, 92, 93; three of these ERCPs were urgent procedures (performed in the patients aged 79, 89, and 92 years old).

In order to test the single variables in their potential association to the onset of complications, we performed univariate logistic regression analyses, but we did not find significant relationships with the outcome (Table 6). Even the type of invasive procedure performed during ERCP was not related with experiencing complications. In particular, unsuccessful procedure (OR 1.45, *p* = 0.437), sphincterotomy (OR 1.14, *p* = 0.630), stenting (OR 0.62, *p* = 0.078), and the combination of sphincterotomy+stenting (OR 1.31, *p* = 0.303) did not relate to the outcome (Table 6).

**Table 6:** Univariate logistic regression analysis of the potential predictors for the onset of ERCP-related complications

Potential risk factors	Odds Ratio	95% Confidence Interval	*p*-value
Age (continuous)	0.99	0.95–1.02	0.399
Age group (≥80)	0.67	0.40–1.12	0.128
Sex, female	0.81	0.50–1.32	0.390
Charlson Comorbidity Index (CCI, continuous)	0.96	0.83–1.10	0.547
Age-adjusted CCI (continuous)	0.93	0.81–1.06	0.260
ASA score (continuous)	1.03	0.64–1.66	0.901
ASA group (III-IV)	1.04	0.59–1.85	0.886
Procedure not completed	1.45	0,57–3.73	0.437
Sphincterotomy	1.14	0.66–1.97	0.630
Stenting	0.62	0.36–1.06	0.078
Sphincterotomy + stent	1.31	0.79–2.18	0.303
Urgency of the procedure	0.79	0.34–1.85	0.584

*ASA* American Society of Anesthesiologists. ASA groups were separated into classes I-II vs. classes III-IV

We also performed a multivariate logistic regression analysis including age (groups of those < 80 vs. those ≥80 years), gender, CCI (groups of those with scores of < 4 vs. those with scores of ≥4), type of intervention (categorical), and urgency of the procedure. Not surprisingly, age, gender, type of procedure and urgency did not result as significantly associated with complications. However, the dichotomized CCI risk scores (in < 4 versus ≥4) resulted as independently and significantly correlated with the outcome (Odds Ratio, OR = 0.29, 95% Confidence Interval, C.I.: 0.09–0.99, *p* = 0.049).

## Discussion

The demand for ERCP is increasing in the elderly and it is important to know the procedure risks and benefits in older patients. The two study groups were different in terms of age, sex, CCI and ASA score.

The older group (ages 80 and above) had a significantly higher number of female patients and this is probably due to the different life expectancy between men and women. This data was recently confirmed in the literature [10].

The older group showed a lower mean CCI, thus raising a question about the existence of a possible selection bias. However, if we consider the age-adjusted CCI, the mean values were significantly higher in older patients. In order to perform ERCP procedures, these data suggest that gastroenterologists specializing in endoscopy probably rely on inclusion criteria that do not take into account the multi-faceted characteristics of the geriatric patient. However, the CCI – age-adjusted or not – should not be considered as a stand-alone assessment tool. Those inclusion criteria often imply that older subjects are selected for their apparent “fitness” according to comorbidity, which is a crucial limitation. Even the ASA physical status classification system - despite being validated for assessing fitness of patients undergoing surgery - cannot be considered as a stand-alone tool for the evaluation of elderly subjects scheduled for ERCP. Not surprisingly, ASA score is mainly a qualitative rather than a quantitative index, theoretically insufficient to describe the complexity of the geriatric population.

A further possible signal of selection bias is the fact that ASA III-IV patients were significantly prevalent in the older group, in potential contrast with the simultaneous presence of a lower index of comorbidity in this age group. Again, even by dichotomizing the CCI in < 4 versus ≥4, the younger group displays a higher number of patients with CCI ≥ 4 than the older one, confirming that patients scheduled for ERCP undergo uneven selection criteria. Those criteria tend to exclude the more comorbid elderly subjects from the procedure, presumably according to the assumption that higher comorbidity translates into higher incidence of complications and mortality related to this procedure.

We agree that several older patients are able to tolerate ERCP, and that older people with comorbidities are at higher risk for some complications compared to those without comorbid conditions,[11] but understanding the “big picture” goes beyond the mere collection of medical history or fitness status. When facing older individuals, a comprehensive geriatric assessment (CGA) should be considered as the preferable way to assess their health status in its entirety. The CGA considers the multiple aspects of the elderly, namely comorbid conditions, polypharmacy, functional level, cognition, and nutritional status among other factors which once combined provide a more reliable prognostic estimation than the ASA score or a comorbidity index alone. We are confident that the role of a CGA will be increasingly relevant for risk stratification and risk management of these patients, or at the very least, a useful guide for avoiding ERCP in subjects who have a high risk of experiencing more harm than benefits from the procedure.

Back to our findings in this study, they were consistent with the literature in terms of indications, diagnoses,[1, 10] and success rates (92.8% vs. 93.5%, for the younger and older group, respectively) [12–15]. The frequencies of ERCP-related complications were 13.2% in the young group and 10.0% in the older, which is slightly higher than the rates reported in the literature (2.5% to 12%). ERCP-related death incidence was 0.7% and 0.5% respectively, in line with previous reports, [12–25] though the only systematic review of ERCPs in the elderly suggests higher rates of death in octogenarians and nonagenarians than in younger patients.[4] We also observed that advancing age was not related to complications, neither in the case of the continuous variable, nor the dichotomized one (OR = 0.99, 95% C.I.: 0.95–1.02, *p* = 0.399; and OR 0.67, 95% C.I. 0.40–1.12, *p* = 0.128, respectively). This is in line with our hypothesis, i.e. that age per se does not carry more complications. However, the finding should still be interpreted carefully because of several limitations.

Neither the indications (given a priori) nor the diagnoses (given *ex-post*, during or after ERCP completion) resulted as significantly related to complications, and again this could be due to the above-mentioned selection bias. Unexpectedly, the type of invasive procedure performed during ERCP was not related with experiencing complications (Table 6).

Pertaining to the multivariate analysis, it should be noted that the sign of the relationship between CCI (dichotomized between < 4 vs. ≥4) and complications is negative, thus it seems that the higher the CCI class, the lower the risk of complications. This fact does not agree with the literature, but it should be interpreted. Indeed, Table 1 showed that a higher number of patients with CCI ≥ 4 belong to the 70–79 age group, and that they do not experience more complications than the older group, opening various interpretations but also confirming the potential impact of the selection bias for this procedure. However, the literature findings do not agree in terms of risk factors for the onset of complications [12–16].

The first limitation of our study is represented by the relatively limited sample size (*n* = 363), though this is similar to that of other studies [3, 23, 26]. The size issue probably explains also part of the difference between findings in the literature. This also gives a possible reason for the absence of significant associations observed at the univariate and multivariate analyses. On the one hand, this is quite unexpected because some of them should be theoretically correlated with complications, e.g. the degree of invasiveness of the procedure (performing sphincterotomy+stent placement is different than an ERCP executed for diagnostic purposes only). On the other hand, those results could be due to the selection bias for this procedure, which is expected to alter the normal correlations, especially if the study population is somewhat limited in size.

In addition, due to the single-centered and retrospective design, our findings cannot be generalized to other acute care settings. Furthermore, we retrieved hospital records but we do not have information about the post-discharge phases of care of the studied ERCP-treated patients. Finally, a major limitation is the lack of a structured, homogenous assessment of the ERCP candidates pre-operatively, which translates to the selection bias enlightened by our analysis. The only study that tried to assess the ERCP-related risk in the elderly population considering multiple domains suggested to include the Duke Activity Status Index (DASI, ranging from 0 to 58.2) to evaluate the functional capacity of subjects with cardiovascular disorders, with higher scores indicating better functional status. Further studies are necessary to confirm the importance of multidimensional scores, i.e. one or more tools able to describe the older patient comprehensively [26].

Given these limitations, this study provides insight and paves the path for including more comprehensive assessment tools in future prospective investigations, which are strongly needed. The final objective is to better characterize the elderly patients pre-operatively and select candidates who will have the highest probability of benefiting from ERCP, reducing the risk of developing intra- and post-operative complications.

## Conclusions

ERCP remains a procedure burdened by many known risks, especially if performed with urgency and in the elderly. Similar to many countries worldwide, the Italian population is progressively aging, [27] and the absolute number of older people that will need ERCP procedures specifically in the future will increase. The possible implementation of a user-friendly and rapid tool for older patients’ assessment will benefit both health professionals as well as their patients, reducing selection biases and improving their risk management. This will potentially and ultimately contribute to contain the incidence of post-ERCP complications and to test the real impact on the patient when the procedure is performed at an advanced age.

## Abbreviations

ASA: American Society of Anesthesiologists
CBD: common bile duct
CCI: Charlson Comorbidity index
CGA: comprehensive geriatric assessment
DASI: Duke Activity Status Index
ERCP: Endoscopic Retrograde Cholangiopancreatography
